# Cardiovascular Risk/Benefit Profile of MHT

**DOI:** 10.3390/medicina55090571

**Published:** 2019-09-06

**Authors:** Paola Villa, Inbal Dona Amar, Maayan Shachor, Clelia Cipolla, Fabio Ingravalle, Giovanni Scambia

**Affiliations:** 1 Department of Obstetrics and Gynecology, Fondazione Policlinico Universitario A. Gemelli IRCCS, UOC di Ginecologia oncologica, 00168 Roma, Italy; 2 Department of Obstetrics and Gynecology, Università Cattolica del Sacro Cuore, 00168 Roma, Italy; 3 Department of Biomedicine and Prevention, University of Tor Vergata, 00133 Rome, Italy

**Keywords:** menopause, menopausal hormone therapy, cardiovascular disease prevention

## Abstract

*Background and Objectives:* Data emerging from the Women’s Health Initiative (WHI) study point toward an association between menopausal hormone therapy (MHT) and cardiovascular (CV) risk. However, post hoc subgroup analyses stratifying participants according to their age and time since menopause, have opened the way to a better understanding of the relationship between estrogen and CV risk. The aim of this review was to revise the current literature and evaluate the CV risk or benefit following administration of MHT considering several factors such as MHT timing, dose, route of administration, and formulation. *Materials and Methods*: An electronic databases search of MEDLINE (PubMed), Cochrane Central Register of Controlled Trials, Web of Science, SCOPUS, congress abstracts, and Grey literature (Google Scholar; British Library) was performed, with the date range from each database’s inception until June 2019. All the studies evaluating MHT and cardiovascular risk, including thromboembolism or stroke, were selected. *Results*: Timing of MHT initiation was shown to be a critical factor in CV risk assessment. In concordance with the “timing hypothesis”, healthy symptomatic women who initiated MHT when aged younger than 60 years, or who were within 10 years of menopause onset, have demonstrated a reduction in both coronary heart disease (CHD) risk and all-cause mortality. In particular, MHT therapy was associated with improvement of subclinical signs of atherosclerosis. Venous thromboembolism (VTE) risk is reduced when low doses of oral estrogen are used. Moreover, transdermal hormonal application significantly reduces CV risk compared with oral administration. MHT impact on the CV system is influenced by either factors inherent to the specific regimen, or factors inherent to the specific patient. Hence, individualization of care is necessary. *Conclusion*: CV risk calculation should be considered by clinicians in order to exclude patients with high CV risk, in whom MHT is contraindicated. Assessing risks and benefits in a patient-centered approach according to individual’s features, health status, and personal preferences is important in order to realize a safe and effective treatment.

## 1. Introduction 

Menopausal hormonal therapy (MHT) ameliorates vasomotor symptomatology, prevents bone loss, and treats estrogen deficiency-related genitourinary symptoms [1]. As such, it is an FDA-approved therapy for these symptoms [1,2]. Whether MHT confers harmful or beneficial effects on cardiovascular (CV) system is controversial. Atherosclerosis and clinical cardiovascular diseases (CVD) tend to appear in women at an older age in comparison with men [3], particularly in the postmenopausal period. Several observational studies have shown that patients taking MHT have reduced CVD risk. Specifically, it was presumed that estrogen serves as a CV protective factor and thus, it was initially assumed that after menopause, MHT can replenish the decline in circulating estrogen and, along with symptomatology relief, can reduce long-term CVD risk [4,5,6,7,8]. Estrogen acts by binding to various receptors and activating multiple signaling pathways [9]. Its protective effects are attributed to serum lipid concentrations modulation [10], as well as estrogen’s direct actions on the vasculature, including vasodilation and inhibition of the response to vascular injury [11]. However, later data emerging from the Women’s Health Initiative (WHI) study [12,13], pointing toward an association between estrogen therapy and CV risk, have challenged this widespread belief and the use of MHT was dramatically reduced [14].

## 2. Materials and Methods

### 2.1. Data Sources

We searched MEDLINE (PubMed), Cochrane Central Register of Controlled Trials, Web of Science, SCOPUS, and Grey literature (Google Scholar; British Library) from January 1980 to June 2019. We used the terms “menopausal hormone replacement therapy”, “hormone replacement therapy”, “estrogen therapy”, “Estrogen, progesterone menopausal therapy”, “Tibolone”, and “T-sec” as text words and as appropriate medical subject headings or equivalent subject headings/thesaurus terms. These terms were combined with “cardiovascular risk”, “thromboembolism”, and “stoke”. The reference lists of all available primary studies were reviewed to identify additional relevant citations.

### 2.2. Screening of Abstract for Eligibility

Abstracts and titles identified from the search were screened by 3 investigators. Disagreements about the inclusion or exclusion of studies were primarily solved by consensus, and when this was not possible, a fourth reviewer resolved them.

### 2.3. Study Selection and Eligibility Criteria

A set of specific criteria were used for selection of literature: Randomized controlled trials (RCT); prospective or retrospective cohort studies; reviews and meta analyses; international societies’ guidelines; studies with outcome measures including cardiovascular risks or benefits; and studies with evaluation of various types of MHT, route of administration, formulation, and dose. Only studies written in English with an available abstract were accepted.

## 3. Results

### 3.1. MHT: Cardiovascular Risk and Benefit Overview

The WHI [12,13] was a large, long-term RCT that enrolled postmenopausal women aged 50–79 years between the years 1993–1998 and was designed to evaluate the risks and benefits of hormonal therapy for the prevention of chronic diseases, including the effects on CVD. The WHI trial randomized women to either combination MHT with estrogen/progestin (conjugated estrogen (CE) 0.625 mg and medroxyprogesterone acetate 2.5 mg/day) or placebo. After a mean follow-up of 5.2 years, this study arm was discontinued due to an increased risk of coronary heart disease (CHD) HR 1.29 (0.85–1.07), stroke HR 1.41 (0.86–2.31), venous thromboembolism (VTE) HR 2.11 (1.26–3.55), and composite CVD risk HR 1.22 (1.09–1.36). An increase in CV events was reported in the extended follow-up post-stopping phase as well [15]. Similarly, no reduction in CHD was shown in the Heart and Estrogen/Progestin Replacement Study (HERS) [16] and the subsequent follow up study (HERSII) [17] in patients with known CHD receiving conjugated equine estrogen and medroxyprogesterone acetate. WHI clinical trials concluded that hormonal therapy does not confer cardiac protection and overall risks in healthy postmenopausal women exceed benefits. 

Some possible factors influencing the negative results of WHI were identified. The trial recruited women with a mean age at enrollment of 63 years, having 12 years mean time since menopause. Only a limited number of women had vasomotor symptoms, many women had CV risk factors at entry (approximately 35% had hypertension), and some had already documented CV events. Moreover, the hormonal doses that were used in the trial are currently considered to be high [18,19].

Thus, it has been stated that the WHI results may not be applied for younger symptomatic menopausal women and/or patients with a more recent onset of menopause. Therefore, post hoc subgroup analyses stratifying participants according to their age and time since menopause were performed [20]. This study noted a trend toward both CHD risk and total mortality reduction in younger women within the first 10 years after menopause. The explanation of these trends has gained the term “the timing hypothesis” [21,22], which states that MHT given in healthy women does not increase CHD risk and possesses the highest cardiovascular benefits when started soon after the onset of menopause. Timing of hormonal therapy initiation was shown to be a critical factor in CV risk assessment, not only in secondary analysis of the WHI study [20,23] but in other studies as well [24,25,26,27,28]. In concordance with the timing hypothesis, a 2015 Cochrane review evaluating randomized controlled trials (RCTs) comparing hormone therapy with placebo demonstrated a reduction in both CHD risk (composite of death from cardiovascular causes and nonfatal myocardial infarction) and all-cause mortality in younger women who had commenced therapy less than 10 years since menopause or were less than 60 years old [29]. In this group of participants, an increased risk of venous thromboembolism was still noted. It is likely that younger women have a healthier heart and less subclinical atherosclerosis at baseline and hence lower CVD risk with hormonal therapy when compared to older women with increased duration of menopause. Indeed, approximately 1000 women aged 50–59 years who participated in the WHI study under the unopposed estrogen treatment arm were evaluated in the WHI Coronary Artery Calcium Study [30]. Computed tomography heart imaging was used to evaluate the coronary artery calcium burden, which was shown to be lower in women assigned to estrogen than in those assigned to placebo (83.1 vs. 123.1; *P* = 0.02). Furthermore, in order to assess MHT effect on atherosclerosis progression, surrogate markers of CVD risk such as measurements of carotid intima-media thickness (CIMT) and Coronary Arterial Calcification (CAC) were done in other important clinical trials [30,31,32].

In the Early vs. Late Intervention Trial with Estradiol (ELITE) study [32], 643 healthy postmenopausal women were grouped according to time since menopause (<6 years past menopause or ≥10 years past menopause). The women were randomly assigned to receive either oral 17 beta-estradiol (1 mg/day plus progesterone 45 mg vaginal gel administered sequentially in women with a uterus) or placebo for a median of five years. This trial has demonstrated that women who received estradiol had lower rates of subclinical atherosclerosis progression, as measured by the rate of change in CIMT, only when therapy was started within 6 years after menopause, but not if started ≥ 10 years after.

The Kronos Early Estrogen Prevention Study (KEEPS) [33,34] has evaluated the hormonal therapy effect on CIMT and CAC as a surrogate to atherosclerosis progression. In the study, 727 healthy women aged 42 to 58 years who were within 3 years after menopause were randomized to treatment arm or placebo. Treatment options were either conjugated equine estrogen (0.45 mg/d) or transdermal estradiol patches (50 mcg/d). Both estrogens were combined with cyclic oral micronized progesterone (200 mg for 12 days each month). No effect on CIMT progression was seen with treatment when compared to placebo. Authors have suggested that these results are due to the short duration of the trial as well as the young age and low risk profile of the participating women [35]. The discrepancy between the ELITE and KEEPS results can be further explained by the low-dose treatment used in the latter (1 mg/d and 0.45 mg/d, respectively) [32,33,34]. Consideration of the WHI subgroups analysis, along with multiple observational studies in the “pre WHI era” and “post WHI era” [36,37] as well as KEEPS and ELITE findings, has opened the way to a better understanding of the relationship between estrogen and CV risk. These data indicate a low-risk, favorable benefit–risk profile for young symptomatic women in early menopause who use MHT in the absence of contraindications [38,39,40,41]. Accordingly, current guidelines [1,42] suggest that benefits are most likely to outweigh risks for healthy symptomatic women who initiate MHT when aged younger than 60 years or who are within 10 years of menopause onset. MHT should not be used for the primary or secondary prevention of coronary heart disease [43,44].

For women aged younger than 60 years or who are within 10 years past menopause onset, considering MHT for menopausal symptom relief, the endocrine society suggest evaluating the baseline risk of CVD and taking this risk into consideration when advising for or against MHT and when selecting type, dose, and route of administration [42]. Risk calculation by modification of the American College of Cardiology (ACC)/American Heart Association (AHA) 10 year CVD risk [45] with stratifications according to years since menopause is available in the Menopause Decision Support Algorithm [46]. Table 1 summarizes the principle statement of current guidelines.

### 3.2. MHT Dose, Route of Administration, and Formulation 

In addition to personal preferences, the individual patient’s characteristics and clinical status might come into play while choosing one route of treatment delivery or specific formulation over another. 

In particular, the dose, estro-progestin/progesterone association, and route of administration must be taken into account.

### 3.3. Dose

The therapeutic goal should be to use the most appropriate, often lowest, effective dose of systemic ET (estrogen therapy) consistent with treatment objectives [1]. A prospective, observational cohort study investigating more than 70,000 healthy postmenopausal women found that MHT is associated with a reduction in major coronary events risk compared with never-users. Importantly, similar reduction was observed with those taking 0.3 mg of oral conjugated estrogen daily and those taking the standard dose of 0.625 mg. However, estrogen at daily doses of 0.625 mg or greater (alone or in combination with progestin) may increase risk for stroke [4]. In addition, while low-dose MHT (such as 0.3 mg conjugated estrogen) was found to have comparable effects on lipoproteins, flow-mediated dilation, and plasminogen activator inhibitor type 1 antigen levels, it had fewer effects on coagulation and inflammatory markers than standard-dose therapy [47]. VTE risk may be reduced with lower doses of oral estrogen than higher doses [48].

### 3.4. Route of Administration

MHT can be administered orally and nonorally (transdermal, vaginal, and intrauterine). Only oral MHT undergoes first-pass metabolism. The first-pass effect can produce benefits including reductions in low-density lipoprotein cholesterol and increases in high-density lipoprotein cholesterol, however, unwanted effects are triglycerides increase and coagulation activation [49,50]. Transdermal administration, by avoiding the hepatic effect, has lower impact on coagulation factors hemostasis [51]. Change in lipid profile was observed in a KEEPS trial [33] in those who had oral conjugated equine estrogens. Studies have demonstrated a lower risk of VTE and stroke with transdermal estrogen (at standard doses) compared with oral [48,52,53,54,55]. For example, a multicenter hospital-based case-control study of postmenopausal women, enrolled 155 consecutive cases with a first documented episode of idiopathic VTE and 381 matching controls. The odds ratio for VTE in current users of oral and transdermal estrogen compared with nonusers was 3.5 (95% CI 1.8–6.8) and 0.9 (0.5–1.6), respectively. Estimated risk for VTE in current users of oral ET compared with transdermal ET users was 4.0 (1.9–8.3). These data suggest that transdermal ET is safer than oral ET regarding thrombotic risk [56]. 

## 4. Formulation

Estrogen alone and estro-progestin administration have different impacts on the cardiovascular risk. Patients who underwent hysterectomy (surgical removal of uterus) for gynecological indications can avoid the administration of progesterone or progestins, which are given in order to decrease the risk of endometrial hyperplasia and subsequent endometrial cancer in women with a uterus. The WHI trials [12,13] have suggested that unopposed estrogen administration have less adverse outcomes in comparison with estrogen plus progestin for treating postmenopausal women. Nevertheless, the knowledge regarding the properties of the different progestins compounds is important. Synthetic medroxyprogesterone acetate is vasoconstrictive, however, natural progesterone possesses vasodilatory properties and might have a positive effect on blood pressure [57,58,59,60]. Drospirenone has antimineralocorticoid diuretic effects and may decrease blood pressure and weight [61,62,63]. Progesterone has a beneficial effect on lipid profile [64]. While non pregnane progestins were associated with excess risk of VTE, no increased risk was noted with medroxyprogesterone acetate and micronized progesterone [65].

Several studies that evaluated the thromboembolic risk of MHT have included separate analyses of estrogen-only and combined estrogen/progestin. However, even if according to available data they confer different risks, it is still inconclusive [66]. 

Furthermore, a 2008 meta-analysis did not observe a difference in the thrombogenicity of estrogen-only versus estrogen/progestin HRT (HR 2.2, 95% CI, 1.6–3.0, and HR 2.6, 95% CI, 2.0–3.2, respectively) [67]. Similarly, LITE investigators calculated similar relative risks for estrogen/progestin (1.6, 95% CI, 1.0–2.6) and estrogen alone (1.6, 95% CI 1.1–2.4) [68]. Likewise, the WISDOM trial did not find a difference in VTE risk between combination and estrogen-only HRT, though it may have been insufficiently powered to do so [69]. On the other hand, a prospective case-control study has demonstrated that estrogen/progestin caused an increased relative risk of 2.7 (95% CI 1.4–5.1) in VTE, whereas estrogen alone did not (RR 1.2, 95% CI 0.6–2.6) [70]. Similarly, another study found that combination hormonal therapy had an odds ratio of 1.6 (95% CI 1.1–2.3) of VTE compared to estrogen-only use [71]. The WHI found an increased risk of VTE in women taking estrogen/progestin replacement [72], but not in those taking estrogens alone [13]. 

### 4.1. Tibolone and Cardiovascular Effects

Tibolone is a synthetic steroid used for the treatment of climacteric symptoms. It is classified as a selective tissue estrogenic activity regulator (STEAR) even though it combines estrogenic, progestogenic, and androgenic activity and has tissue-specific properties [73]. Tibolone has complex effects on lipid profile and, along with beneficial effects on a few cardiovascular markers (total cholesterol, LDL, lipoprotein (a) levels), it seems to decrease HDL cholesterol [74,75,76,77]. Moreover, it is associated with C-reactive protein elevation and modulation of other inflammatory markers [77,78]. Despite these features, tibolone appears to exert antiatherogenic properties and was found to reduce atherosclerosis progression in animals [79,80] and in humans [81,82]. VTE risk does not increase with tibolone [83,84,85]. Nevertheless, tibolone may increase the risk of stroke in older women [84,86]. 

In a randomized study designed to test the tibolone efficacy on bone metabolism, 4538 osteoporotic women, who were between the ages of 60 and 85 years received once-daily tibolone (at a dose of 1.25 mg) or placebo. Tibolone reduced the risk of fracture and breast cancer, but increased the risk of stroke in these older women. The overall number of adverse events was small, and no increased risk of venous thromboembolism or coronary events was observed [84].

### 4.2. Tissue-Selective Estrogen Complex (TSEC) and Cardiovascular Effects

Conjugated estrogen-bazedoxifene (CE/BZA) is currently the only approved TSEC for managing VMS and osteoporosis prevention in postmenopausal women. BZA, a selective estrogen receptor modulator (SERM), protects against estrogenic effects on the uterus and thus may be used for non-hysterectomized women without the need for progestin administration. 

Most of the evidence that has led to CE/BZA approval is derived from a series of phase III trials named SMART (selective estrogens, menopause, and response to therapy) [87,88,89,90]. Cardiovascular safety of CE/BZA was assessed by a meta-analysis of five SMART trials [91]. Rates of stroke, VTE, and CHD-related events were comparable to placebo in healthy postmenopausal women who were given at least one dose of CE 0.45 or CE 0.625 mg with BZA 20 mg for up to two years. However, BZA effect on venous thromboembolism risk remain unclear, especially because a previous five-year phase III study on BZA alone did show more frequent VTE [92]. Another pooled analysis of three SMART trials reported mostly favorable changes in lipid parameters (total cholesterol, LDL, HDL), while triglycerides were significantly increased compared to placebo [93]. 

## 5. Conclusions

MHT impact on the CV system is influenced by either factors inherent to the specific regimen (route of administration, formulation, dose, and duration of use) or factors inherent to the specific patient (other CV risk factors, comorbidities, and age at menopause onset and at therapy initiation). Hence, individualization of care is necessary. Healthy symptomatic postmenopausal women should initiate MHT when aged younger than 60 years or who are within 10 years of menopause onset. MHT should be prescribed at the lowest effective dose and the transdermal route is preferred in some cases. A clinician should consider the risks and benefits in a patient-centered approach, assessing the individual’s features, health status, and preferences.

## Figures and Tables

**Table 1:** Data summarized from North American Menopause Society 2017 guidelines [1] and Endocrine Society Clinical Practice Guideline [42].

Coronary Heart Disease (CHD)	Venous Thromboembolism (VTE)	Stroke	Mortality
- Menopausal hormone therapy (MHT) is a safe and effective treatment option of menopausal symptoms when introduced in healthy postmenopausal women that are within 10 years of menopause onset or aged younger than 60 years; some data suggest reduced risk of CHD in this age range. - It is important to evaluate the baseline risk of cardiovascular disease (CVD) and to consider this risk when advising for or against MHT and when selecting type, dose, and route of administration - For women at high risk of CVD, MHT is not recommended. -For women with moderate risk of CVD, transdermal estradiol should be offered as first-line treatment, alone for women without a uterus, or combined with micronized progesterone (or another progestogen that does not adversely modify metabolic parameters) for women with a uterus. - In women who start using MHT more than 10 years from menopause onset, and evidently by 20 years, there is potential for increased risk of CHD. -Hormone therapy is not FDA indicated for primary or secondary cardioprotection.	- For women at increased risk of VTE who request MHT, a nonoral route of therapy at the lowest effective dose is recommended, if not contraindicated; -For women with a uterus, we recommend a progestogen (for ex. progesterone and dydrogestone) that is neutral on coagulation parameters.	- A meta-analysis of randomized controlled trials (RCTs) of women who initiate MHT found no increased risk of stroke in women aged younger than 60 years or who were within 10 years of menopause onset, whereas observational study findings are mixed. - Lower-dose oral as well as lower-dose transdermal therapy has less effect on risk of stroke, compared with standard-dose oral MHT *	Meta-analyses of RCTs report a significant reduction in all-cause mortality in women who initiate MHT when aged younger than 60 years and/or are within 10 years from menopause onset. - No protective effect was found in women with initiation more than 10 years from menopause onset.
Personal and familial risk of CVD, stroke, and VTE should be considered when initiating MHT.			

* Evidence based on observational studies and meta-analyses, although RCT data are still lacking.
